# Arthroscopic Reduction and Fixation of Impacted Humeral Head Reverse Hill‐Sachs Fracture With Absorbable Anchors for Acute Traumatic Posterior Shoulder Dislocation

**DOI:** 10.1002/atn2.70277

**Published:** 2026-10-08

**Authors:** Yuxin Lin, Chuanqiang Liu, Jiandong Gao, Jiushan Yang, Shaoshan Wang, Lizhong Jing

**Affiliations:** ^1^ Shandong University of Traditional Chinese Medicine Jinan Shandong China; ^2^ Department of Sport Medicine Shandong University of Traditional Chinese Medicine Affiliated Hospital Jinan Shandong China; ^3^ Department of Radiology Shandong University of Traditional Chinese Medicine Affiliated Hospital Jinan Shandong China

## Abstract

A minimally invasive technique is presented for the treatment of acute traumatic posterior shoulder dislocation with a reverse Hill‐Sachs lesion. Arthroscopic reduction was combined with internal fixation using 1 absorbable anchor. Under high‐definition arthroscopic visualization, precise anatomical reduction of the impacted anteromedial humeral head compression fracture was achieved. Absorbable anchors were inserted into the subchondral bone defect beneath the reduced fragment. Initial stability was provided, and congruity of the articular surface is maintained. Compared with conventional approaches, several advantages were shown. First, anatomical restoration and functional preservation were achieved. The osseous morphology of the humeral head was restored, and the subscapularis tendon was preserved. Postoperative recovery of shoulder function, particularly internal rotation, was facilitated. Second, minimal invasiveness and biocompatibility were emphasized. Tissue trauma was reduced, and visualization was improved. Mechanical support was provided during early healing, followed by gradual degradation of the implants and progressive load transfer to newly formed bone. Long‐term risks associated with metallic implants were avoided, including secondary removal and stress shielding. Finally, concomitant injuries could be managed concurrently under arthroscopic guidance.

## Graphical abstract

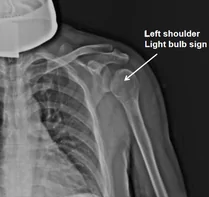

**VIDEO 1:** Arthroscopic reduction and internal fixation of an impacted reverse Hill–Sachs fracture using an absorbable anchor in the left shoulder. Video content can be viewed at https://doi.org/10.1002/atn2.70277.

The shoulder joint is the most commonly dislocated joint in the body, yet posterior shoulder dislocation is rare, accounting for less than 4% of all shoulder dislocations. Because of its low incidence, the rate of missed diagnosis exceeds 50%.^1^ Common causes include seizures, trauma, and electric shock.^2^ Cases have also been reported in athletes subjected to high‐intensity posterior‐loading activities and in individuals exposed to repetitive microtrauma during military training.^3^ Typical posterior shoulder dislocations are often associated with a reverse Bankart lesion or a reverse Hill‐Sachs lesion.^4^ They may also occur alongside a McLaughlin lesion, glenoid rim fractures, humeral fractures, nerve injuries, and osteochondral defects, whereas rotator cuff injuries are rarely observed.^5^ Diagnosis and treatment selection remain clinically challenging because of heterogeneous injury mechanisms and patterns.

The management of posterior shoulder dislocation depends on the size of the humeral head defect, degree of instability, duration of dislocation, and the patient's functional demands. For acute traumatic posterior shoulder dislocation with a reverse Hill‐Sachs fracture, surgical intervention is generally the most reliable approach. Several surgical techniques have been described, each with specific indications and limitations (see the Discussion section). In the present case, arthroscopic reduction of the reverse Hill‐Sachs fracture was performed, followed by the placement of composite absorbable anchors deep to the reduced fragment to maintain fragment height. This technique is indicated for acute traumatic posterior shoulder dislocation with a reverse Hill‐Sachs defect involving less than 25% of the articular surface, provided that the impacted fragment and the contralateral hinge remain intact and that no concomitant fractures of the glenoid, proximal humerus, or other sites requiring urgent fixation are present. According to the classification proposed by Paksoy et al., the present case is categorized as type A2.^6^

## SURGICAL TECHNIQUE

Preoperative radiographic and computed tomography findings in the present case are shown in Figures 1 and 2, respectively. After general anesthesia, the patient is positioned in the lateral decubitus position. The elbow is wrapped in a sterile drape and placed in approximately 45° of abduction under longitudinal traction. Key landmarks, including the acromion, coracoid process, and acromioclavicular joint, are marked, and standard posterior, anterosuperior, and anteroinferior portals are established.

**FIGURE 1:**
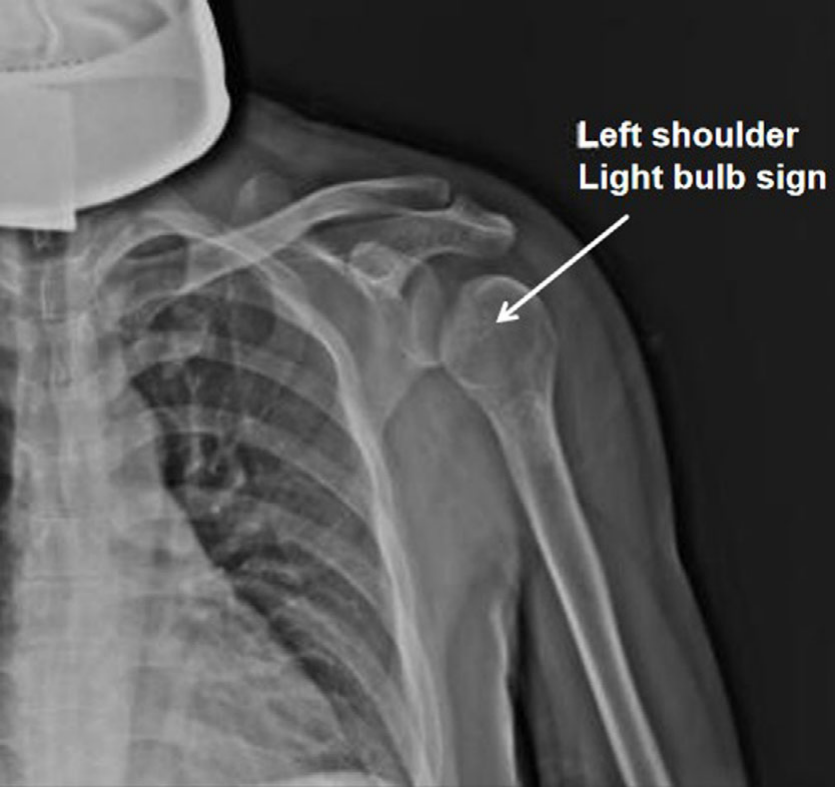
Anteroposterior radiograph showing a left posterior shoulder dislocation with the characteristic “light bulb sign.”

**FIGURE 2:**
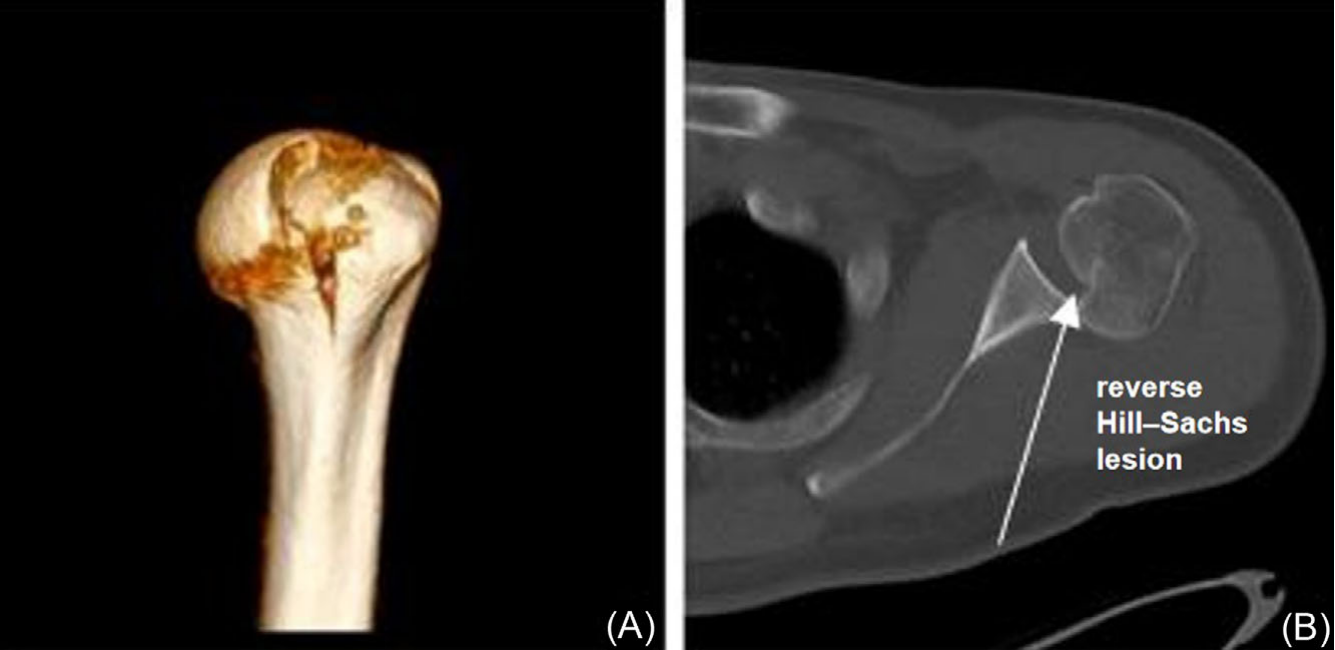
(A) CT with 3‐dimensional reconstruction and (B) horizontal‐level CT images reveal the reverse Hill‐Sachs lesion (arrow) and the resulting humeral head defect. (CT, computed tomography.)

The posterior portal, created approximately 1.5 cm inferior and 1.5 cm medial to the posterolateral edge of the acromion, is used for arthroscope insertion. The anterosuperior and anteroinferior portals are established using an outside‐in technique. The anterosuperior portal is positioned posterior to the biceps tendon within the plane of the glenohumeral joint, whereas the anteroinferior portal is placed at the superior border of the subscapularis tendon. Threaded cannulas are inserted, and diagnostic arthroscopy is performed to evaluate the posterior capsulolabral complex, glenoid, the long head of the biceps tendon, rotator cuff, and humeral head, with specific attention to the reverse Hill‐Sachs lesion.

Arthroscopic examination revealed no significant abnormalities of the long head of the biceps tendon or rotator cuff. The reverse Hill‐Sachs fracture site is exposed (Figure 3A), measuring approximately 6 mm in depth and 1 cm in width. The impacted fragment is depressed but intact, with the contralateral hinge being preserved. A fresh tear of the posterior labrum and capsule with deep displacement is also noted.

**FIGURE 3:**
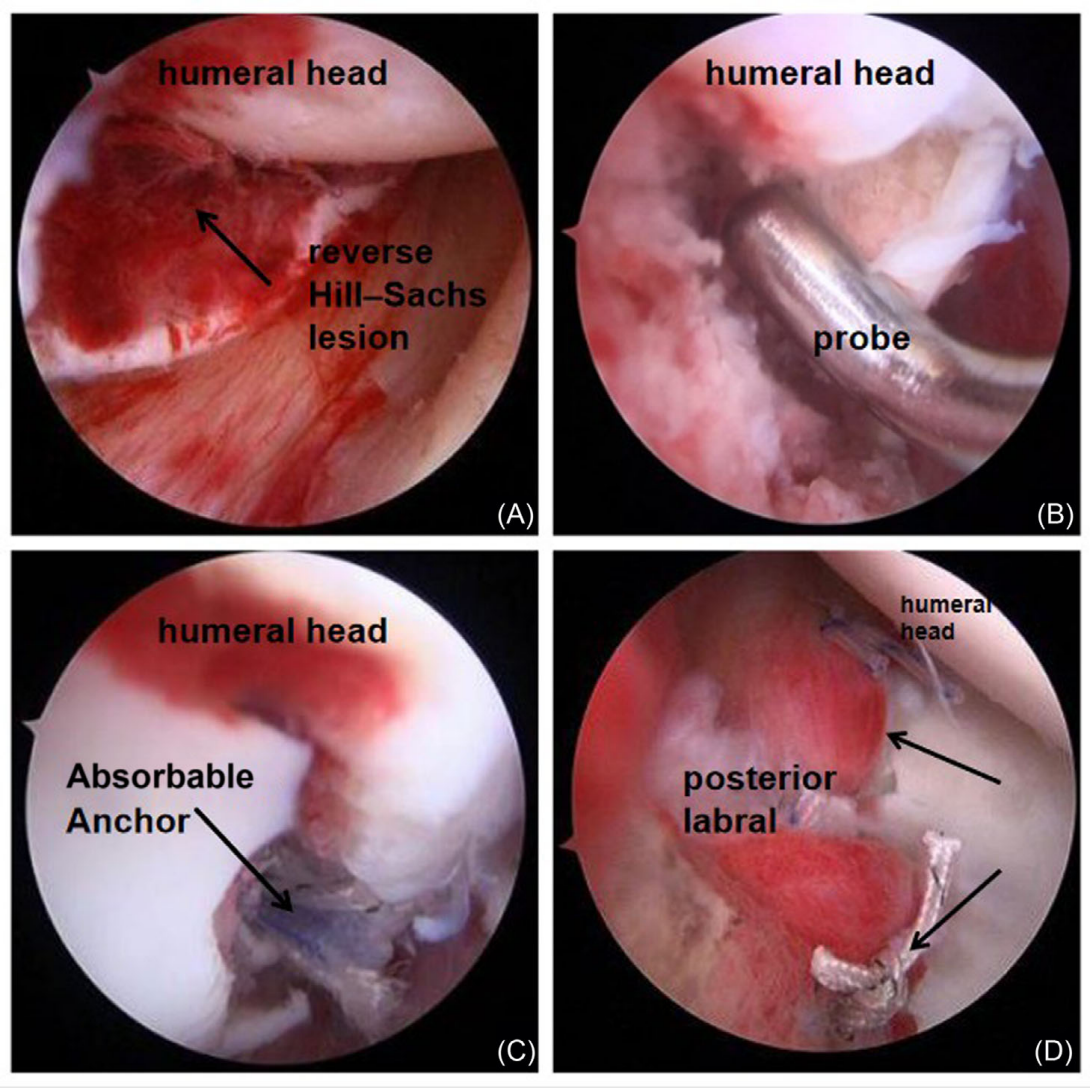
With the patient in lateral decubitus position, arthroscopy of the left shoulder is performed through the anterosuperior portal. (A) Exposing the impacted reverse Hill‐Sachs fracture area; (B) reduction of the fracture fragment using a probe, restoring near‐anatomical height; (C) an absorbable anchor deep to the reduced fragment to maintain its position; and (D) final arthroscopic view after repair of the torn posterior labrum using 2 all‐suture anchors.

Under visualization from the anterosuperior portal, a probe is introduced through the anteroinferior portal and carefully inserted into the gap between the fracture ends. The fracture fragment is gently elevated and reduced. Near‐anatomical height is restored (Figure 3B). To maintain reduction, 1 absorbable anchor (Suture Anchor, Arthrex, Naples, FL) is inserted into the void beneath the reduced fragment (Figure 3C). Sutures are trimmed with an arthroscopic suture cutter, rather than withdrawn, to preserve anchor stability. Finally, 2 all‐suture anchors (Y‐Knot All‐Suture Anchor, CONMED, Largo, FL) are used to repair the torn posterior labral tissue (Figure 3D). The detailed surgical procedure is shown in Video 1.

### Postoperative Management

Postoperative computed tomography with 3‐dimensional reconstruction confirmed satisfactory reduction of the reverse Hill‐Sachs fracture and restoration of the humeral head contour (Figure 4A and D). During the initial postoperative period, the shoulder was immobilized in a brace positioned in slight external rotation (approximately 15°‐30°) and abduction (approximately 15°) for 1 month. Elbow flexion‐extension and grip‐strength exercises were permitted during this period. Active shoulder mobilization was initiated at 6 weeks, whereas resisted exercises were restricted for the first 3 months.

**FIGURE 4:**
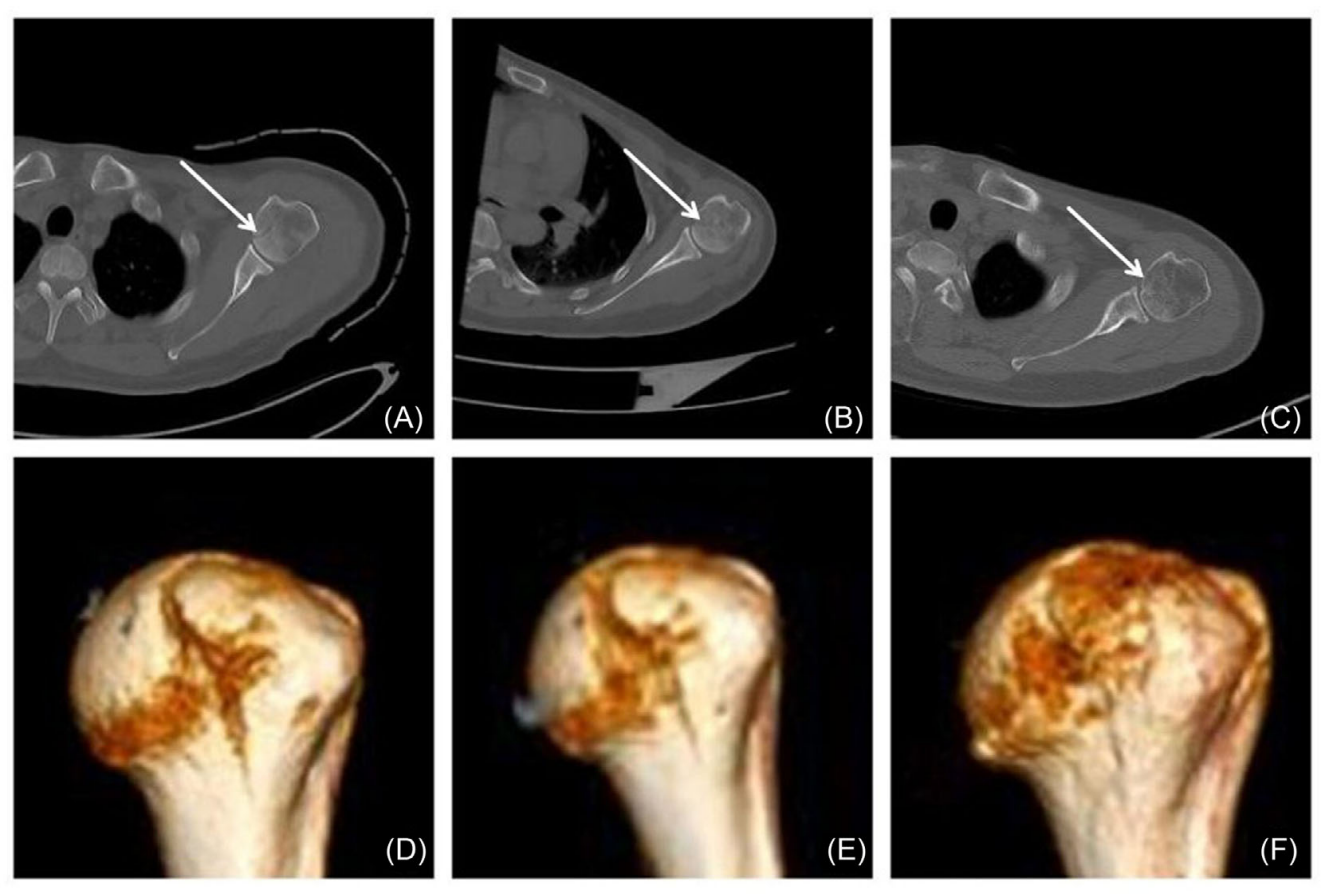
Postoperative axial CT scans (A) immediately after surgery, (B) at 1‐month follow‐up, and (C) at 3‐month follow‐up, showing progressive consolidation and healing of the reduced fracture fragment. Three‐dimensional CT reconstructions of the humeral head (D) immediately postoperatively, (E) at 1 month, and (F) at 3 months, illustrating the maintained reduction and contour restoration over time. (CT, computed tomography.)

## DISCUSSION

The management of acute traumatic posterior shoulder dislocation focuses on prompt and safe closed reduction of the glenohumeral joint, followed by accurate assessment and definitive treatment of the reverse Hill‐Sachs lesion, with treatment selection depending on humeral head defect size. Recent literature has similarly emphasized that treatment selection should be guided by defect size and joint stability, with no clear consensus regarding the optimal surgical strategy.^7^ For defects <20% of the articular surface, closed reduction achieves stability; conservative management with strict bracing and phased rehabilitation (balancing joint protection and stiffness prevention) suffices. For 20% to 40% defects, recurrent dislocation risk increases, requiring surgical intervention (historically, McLaughlin procedure and modifications to transfer the subscapularis tendon). For defects >40%, extensive articular loss demands complex reconstruction (open reduction with bone grafting and rotational osteotomy) or shoulder arthroplasty.^8^

However, traditional techniques have limitations. Allogeneic bone transplantation is the only method restoring the original joint surface^9^ but carries risks of graft collapse, degeneration, limited availability, and high cost.^3^, ^7^, ^10^, ^11^ In contrast, fixation using absorbable anchors represents a conceptual shift: rather than replacing bone, it supports and guides endogenous regeneration. This approach offers several theoretical and practical advantages. It provides a reliable, off‐the‐shelf solution while avoiding donor‐site morbidity and limitations related to allograft procurement. Costs are generally predictable and often lower than those of structural allografts. Mechanically, the anchors offer stable support during the critical healing phase, whereas, biologically, they facilitate new bone formation. Because the anchors gradually degrade, load is progressively transferred to the regenerated bone, meeting the healing requirements. Additionally, long‐term risks associated with metallic implants, such as osteolysis and stress shielding, are avoided.

The traditional McLaughlin procedure alters shoulder anatomy, impairs internal rotation, and is unsuitable for large defects.^12^, ^13^, ^14^, ^15^, ^16^ Our technique addresses the lesion while preserving the subscapularis tendon, in contrast to modified McLaughlin procedures that rely on tendon transfer to fill the defect.^17^ The reverse Hill‐Sachs defect is reduced without violating the subscapularis using a levering method, and subchondral support is provided through deep anchor placement. This approach achieves anatomical reduction while maximizing soft‐tissue integrity, maintaining the native rotator cuff force couple and glenohumeral kinematics, and establishing a foundation for postoperative functional recovery. Banerjee et al.^18^ conducted a retrospective analysis of 7 patients with acute posterior shoulder dislocations and large humeral head defects (25%‐45%) who underwent a modified McLaughlin procedure within 14 days postinjury. At 3‐year follow‐up, patients achieved excellent average ranges of motion: 175.7° of forward flexion, 171.4° of external rotation at 90° abduction, and 83.6° of external rotation. However, a key outcome, internal rotation, was not reported despite its dependence on an intact and properly tensioned subscapularis tendon. In the present case, comparable recovery of overall shoulder mobility was achieved, with both internal rotation strength and range preserved. This favorable outcome is attributed to the preservation of the subscapularis tendon and anatomical restoration of the articular surface.

Marco et al.'s^19^ cortical window technique enables anatomical reduction but risks poor visualization and secondary fracture. Arthroscopic surgery offers better prognosis than open surgery.^20^ Arthroscopy combines precise manipulation with minimally invasive treatment. High‐definition arthroscopy offers a panoramic, magnified, and dynamic view of the glenohumeral joint and associated defects. Fracture morphology, cartilage integrity, and soft‐tissue attachments are clearly visualized, allowing reduction under direct observation. Fixation can be performed under dynamic assessment, including probing and range‐of‐motion testing, enhancing both fracture alignment accuracy and fixation stability. The use of minimally invasive portals reduces injury to surrounding neurovascular structures and minimizes postoperative scarring, thereby creating favorable conditions for improved functional outcomes (Tables 1 and 2).

**TABLE 1:** Summary of Advantages and Limitations of the Described Arthroscopic Technique

**Advantages**	**Limitations**
1. Achieves anatomical reduction of the humeral head	1. Not applicable for comminuted reverse Hill‐Sachs fractures or those with an incompetent contralateral hinge
2. Preserves the anatomical integrity of the lesser tuberosity and subscapularis tendon	2. Not suitable for cases with concomitant major fractures of other structures requiring fixation
3. Allows for simultaneous arthroscopic management of associated intra‐articular pathologies (e.g., glenoid labral tears, rotator cuff injuries)	3. May be less suitable for patients with larger humeral head defects (>25%‐40% of articular surface)
4. Minimally invasive procedure with relatively simple operative steps	4. Requires a high level of technical proficiency in arthroscopic surgery
5. Provides excellent, magnified visualization of the joint and pathology	
6. Enables restoration of a full, functional range of shoulder motion with systematic rehabilitation	

**TABLE 2:** Pearls and Pitfalls

**Pearls**	**Pitfalls**
1. The fracture fragment is gently elevated to avoid fracture of the hinge	1. Make sure the impacted fragment is depressed but intact, with the contralateral hinge preserved
2. To maintain reduction, 1 absorbable anchor (or more) is inserted into the void beneath the reduced fragment	
3. Sutures are trimmed with an arthroscopic suture cutter, rather than withdrawn	

Nevertheless, certain limitations of our technique warrant consideration. The void beneath the reduced bone lacks osseous support (potential cyst formation). Efficacy for defects >25% is unproven. The procedure is unsuitable for concomitant fractures.

In conclusion, arthroscopic reduction and internal fixation with absorbable anchors are reliable options for acute traumatic posterior shoulder dislocation with reverse Hill‐Sachs lesion under strict indications.

## DECLARATION OF GENERATIVE AI AND AI‐ASSISTED TECHNOLOGIES IN THE WRITING PROCESS

During the preparation of this work, the authors did not use AI and AI‐assisted technologies in the writing process.

## DISCLOSURES

The authors (Y.L., C.L., J.G., J.Y., S.W., L.J.) declare that they have no known competing financial interests or personal relationships that could have appeared to influence the work reported in this article.
